# Preparation and Adsorption Properties of MCM-41 with Novel Gemini Ionic Liquid Surfactants as Template

**DOI:** 10.3390/ma15082780

**Published:** 2022-04-10

**Authors:** Xuzhao Yang, Yarong Bai, Qing Li, Jun Wang

**Affiliations:** Zhengzhou Key Laboratory of Fine Chemicals, School of Material and Chemical Engineering, Zhengzhou University of Light Industry, Zhengzhou 450001, China; 14747149958@163.com (Y.B.); lq13461536442@163.com (Q.L.); wangjun8828@sina.com (J.W.)

**Keywords:** Gemini ionic liquid surfactants, mesoporous molecular sieves, crystal violet, thermodynamics, kinetics

## Abstract

A mesoporous molecular sieve was prepared by the hydrothermal synthesis method with symmetric Gemini surfactant 1,3-bis(hexadecyldimethylammonio)-propane dibromide, symmetric Gemini ionic liquid surfactant 1,3-bis(3-hexadecylimidazolium-1-yl) propane dibromide and self-designed asymmetric Gemini ionic liquid surfactant 1-(3-(hexadecyldimethylammonio)prop-1-yl)-3-hexadecylimidazolium dibromide as the template agent. The structure characterization results for mesoporous molecular sieves show that the material possesses a hexagonal pore structure with uniform channels. The mesoporous silica that was synthesized with self-designed asymmetric Gemini ionic liquid surfactant as the template agent possesses the largest surface area and its pore size and specific surface area are, respectively, 3.28 nm and 879.37 m^2^/g. The adsorption properties of the prepared MCM-41 for crystal violet were investigated, and the adsorption thermodynamics and kinetics were investigated. The results show that adsorption equilibrium can be reached under pH = 9 and 35 °C for 50 min, and the quantity of adsorption can reach up to 464.21 mg/g. The adsorption process belongs to Langmuir isothermal adsorption, conforming to second-order adsorption kinetics, and the adsorption process is an endothermic process.

## 1. Introduction

MCM-41 mesoporous molecular sieves can be used under harsh reaction conditions. This is because of their desirable characteristics such as highly ordered mesoporous structure, high surface area and other unique characteristics [1,2]. The first surfactant used in the fabrication of MCM-41 was cetyltrimethylammonium chloride [3]. In recent years, Gemini surfactants have been used for the fabrication of mesoporous materials. For example, Zhao et al. [4] successfully synthesized ultramicroporous silica with a hexagonal pore structure using the Gemini cationic surfactant [N_1116_C_2_N_1116_][Br]_2_ as a templating agent, showing a specific surface area of 795 m^2^/g. Voort et al. [5] successfully prepared MCM-41 under alkaline conditions by using [N_1116_C_8_N_1116_][Br]_2_ with a long connecting base as the templating agent. G. Berlier et al. [6] synthesized ordered mesoporous MCM-41 at room temperature with Gemini surfactants as structure directing agents. Wang et al. [7] studied the relationship between the intermediate connecting chain of Gemini surfactant and the pore size of mesoporous silica material, and the pore size of silica material decreased significantly with the increase in the connecting chain length of Gemini surfactant. Li et al. [8] prepared a radial-orientation hollow mesoporous silica material using Gemini surfactant [N_1114_C_2_N_1114_][Br]_2_ as template and tetraethylorthosilicate as the silicon source. Traditional cationic surfactants can be used in the fabrication of mesoporous materials due to their amphiphilic properties. As a new type of cationic surfactant, ionic liquid surfactants have a wider range of structural design and performance regulation than conventional surfactants. Moreover, regarding Gemini ionic liquid surfactants as a new class of ionic liquids, their molecular structure contains a covalently connected double cation and two separate anions, and shows the unique properties of ionic liquids, such as ultra-low surface tension, adjustable structure and so on. Using Gemini ionic liquid surfactant as a template agent, the structure of mesoporous materials can be controlled not only by changing the synthesis conditions but also by changing the length of the hydrophobic chain and the bonding group [5,9]. Meanwhile, its critical micelle concentration is 1–2 orders of magnitude lower than that of traditional surfactants, which makes it easier to form aggregates as soft templates of mesoporous molecular sieves in solution. To our knowledge, the application of asymmetric Gemini ionic liquid surfactants in mesoporous materials has not yet been reported elsewhere.

Recently, mesoporous molecular sieves have attracted increased attention in organic dye wastewater treatment [10,11,12,13,14]. Lee et al. [10] studied the adsorption of crystal violet on MCM-41, and the maximum adsorption capacity was 216.65 mg/g. Li et al. [15] prepared an MCM-41 mesoporous molecular sieve and studied its adsorption effect on the alkaline dye crystal violet in water, with an adsorption capacity of 436.21 mg/g. An et al. [16] found that MCM-41 had a high removal rate of methylene blue at room temperature. The imidazolium in the imidazolyl-containing Gemini ionic liquid surfactant has strong polarity, which gives it stronger aggregation ability, which is more conducive to the formation of mesoporous structure and involves less usage.

Therefore, this work focused on mesoporous materials which were synthesized with a self-designed asymmetric Gemini ionic liquid surfactant 1-(3-(hexadecyldimethylammonio)prop-1-yl)-3-hexadecylimidazolium dibromide ([N_1116_C_3_IMC_16_][Br]_2_), a symmetric Gemini ionic liquid surfactant 1,3-bis(3-hexadecylimidazolium-1-yl) propane dibromide ([C_16_IMC_3_C_16_IM][Br]_2_), and a traditional Gemini surfactant 1,3-bis(hexadecyldimethylammonio)-propane dibromide ([N_1116_C_3_N_1116_][Br]_2_) as the templating agent. The structural characterization of the mesoporous materials obtained from [N_1116_C_3_IMC_16_][Br]_2_ was analyzed and the schematic diagram of the preparation of mesoporous molecular sieve with Gemini ionic liquid surfactant as a templating agent is presented. The adsorption properties of the organic dye crystal violet in water were systematically investigated, together with the adsorption thermodynamics and kinetics, laying a theoretical foundation for the in-depth understanding of the treatment of organic dyes with mesoporous molecular sieves.

## 2. Materials and Methods

### 2.1. Materials

For the synthesis of surfactant and mesoporous molecular sieves, the following standard compounds were used: imidazole (Shanghai Macklin Biochemical Co., Shanghai, China, ≥99.0%), 1,3-dibromopropane (Shanghai Macklin Biochemical Co., Shanghai, China, ≥99.0%), hexadecyltrimethylammonium bromide (Shanghai Aladdin Biochemical Technology Co., Ltd., Shanghai, China ≥98.0%), bromohexadecane (Shanghai Macklin Biochemical Co., Shanghai, China, 98.0%), acrylonitrile (Shanghai Aladdin Biochemical Technology Co., Ltd., Shanghai, China, ≥99.5%), ethyl acetate (Shanghai Macklin Biochemical Co., Shanghai, China, ≥99.5%), tetraethylorthosilicate (Shanghai Aladdin Biochemical Technology Co., Ltd., Shanghai, China, 98.0%) and crystal (Shanghai Aladdin Biochemical Technology Co., Ltd., Shanghai, China, ≥90.0%), ammonia (Shanghai Macklin Biochemical Co., Shanghai, China, 25%), anhydrous ethanol (Shanghai Aladdin Biochemical Technology Co., Ltd., Shanghai, China, ≥99.5%), isopropanol (Shanghai Aladdin Biochemical Technology Co., Ltd., Shanghai, China, ≥99.5%) and methanol (Shanghai Aladdin Biochemical Technology Co., Ltd., Shanghai, China, ≥99.5%). All experimental reagents were used directly without further purification.

### 2.2. Synthesis and Characterization of Surfactants

**Figure 1:**
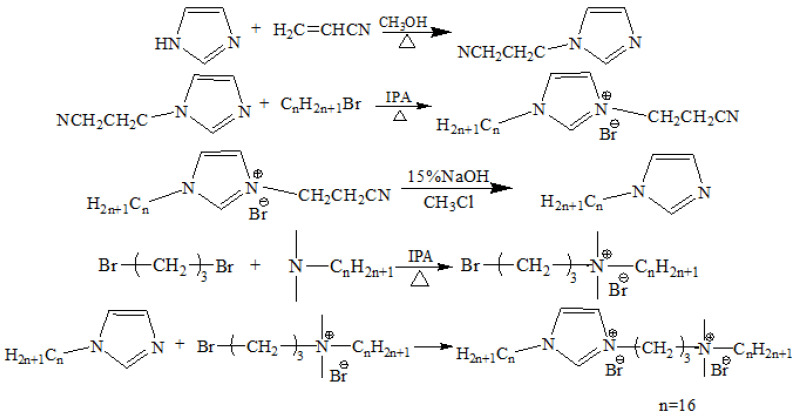
Schematic diagram of the synthesis steps of [N_1116_C_3_IMC_16_][Br]_2_.

Asymmetric Gemini ionic liquid surfactant [N_1116_C_3_IMC_16_][Br]_2_ was designed and prepared according to the following procedure using N-hexadalkylimidazole and *N*,*N*-dimethyl (1-bromopropyl) alkyl bromide as raw materials (Figure 1). The intermediate N-alkylimidazole was synthesized in a three-step procedure by reacting imidazole with acrylonitrile and long-chain bromoalkene. We added some quantities of imidazole and acrylonitrile to a round-bottom flask, with anhydrous methanol as the solvent, and the reaction was carried out at 50 °C for 8 h and then cooled to 40 °C. The unreacted acrylonitrile was removed by rotary evaporation, and the crude product was vacuum dried to obtain N-nitrile ethylimidazole. Then, N-nitrile ethylimidazole and bromoalkane were dissolved into isopropanol, and the reaction was carried out at 50 °C for 18 h. Isopropanol was then removed by rotary evaporation to obtain the intermediate product 1-nitrile ethyl-3-alkylimidazole bromide. Fifteen percent NaOH solution and chloroform were mixed with 1-nitrile ethyl-3-alkylimidazolium bromide, and the mixture was continuously stirred at room temperature until the reaction was complete. The obtained mixture was separated, and the lower layer of brown liquid was washed with double distilled water to obtain the brown viscous liquid N-alkylimidazole. The yield was 89.7%, and the product mass fraction was 99.5%. Using tetrabutylammonium bromide as a catalyst, a certain amount of *N*,*N*-dimethylalkyl tertiary amine and excess 1,3-dibromopropane were added to isopropanol, and the reaction was carried out at 40 °C for 10 h. Isopropanol was removed by rotary evaporation. The obtained white solid was washed with ethyl acetate and dried in vacuo to obtain a white powder *N*,*N*-dimethyl(1-bromopropyl) alkyl ammonium bromide (yield 83.2%, mass fraction 99.1%). A certain amount of *N*-alkylimidazole and *N*,*N*-dimethyl(1-bromopropyl)alkylammonium bromide were added to isopropanol and reacted at 80 °C for 72 h. The isopropanol was removed and the product, [N_1116_C_3_IMC_16_][Br]_2_, was obtained (yield 88.6%, mass fraction 99.7%). The chemical structure (^1^H NMR and ^13^C NMR) of [N_1116_C_3_IMC_16_][Br]_2_ was characterized by NMR spectroscopy (Bruker Advance NEO 600 spectrometers, Bremen, Germany) with dimethyl sulfoxide-*d_6_* (DMSO- *d_6_*) as the solvent.

**Figure 2:**
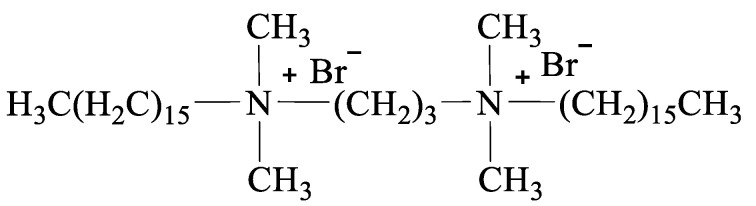
The molecular structure of [N_1116_C_3_N_1116_][Br]_2_.

**Figure 3:**
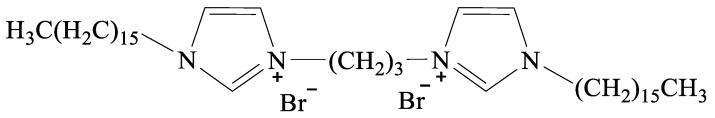
The molecular structure of [C_16_IMC_3_C_16_IM][Br]_2_.

Symmetrical Gemini cationic surfactants [N_1116_C_3_N_1116_][Br]_2_ and [C_16_IMC_3_C_16_IM][Br]_2_ were prepared according to the method reported in the literature [17] and their chemical structures are presented in Figure 2 and Figure 3. The yield and mass fraction of [N_1116_C_3_N_1116_][Br]_2_ were, respectively, 89.6% and 99.6%, and those for [C_16_IMC_3_C_16_IM][Br]_2_ were 87.6% and 99.5%, respectively.

### 2.3. Preparation and Characterization of Mesoporous Silica

Using the above ionic liquid surfactants as template agents, a certain amount of ionic liquid was dissolved in water at 40 °C, then ammonia was added to adjust the pH value above 10, and 2.08 g tetraethylorthosilicate was dropped and stirred for 12 h. The reactants were moved into a Teflon reactor and heated at 100 °C for 24 h, and the resulting products were washed to the filtrate to be neutral. After drying, white powder was obtained by calcination at 550 °C for 12 h. The amounts of reactants and substances corresponding to different templates are given in Table 1. 

The mesoporous silica was characterized by wide-angle Fourier transform infrared spectroscopy (FTIR), X-ray photoelectron spectrometry (XPS), X-ray diffraction (XRD), nitrogen adsorption-desorption isotherms and scanning and transmission electron microscopy. FTIR spectra were obtained using an FTIR spectrometer (Bruker INVENIO, Karlsruhe, Germany). The spectra were collected at 32 scans with a spectral resolution of 2 cm^−1^ within the wave number range 400 to 4000 cm^−1^. Surface chemical analysis was performed using an XPS (Thermo VGESCALAB 250, Waltham, MA, USA) fitted with an Al monochromatic X-ray source (1486.6 eV). An electron flood gun was used to compensate for static charge build up. Elemental atomic concentrations were calculated from the XPS peak areas and the corresponding Scofield sensitivity factors corrected for the analyzer transmission work function. X-ray diffraction (XRD) patterns were obtained using an X-ray diffractometer (D8 ADVANCE, Karlsruhe, Germany). XRD data were collected in a scan mode with the scanning speed of 0.5°/min in the 2*θ* range between 1° and 10° at ambient temperature. N_2_ adsorption desorption isotherms were acquired using a BELSORP-miniII (Microtrac BEL, Tokyo, Japan) apparatus. A surface area analyzer (Micromeritics ASAP Tristar, Tokyo, Japan) was used to measure gas adsorption isotherms. The sample was pre-treated under nitrogen gas at 150°C for a period of 10 h. The Brunauer–Emmett–Teller (BET) method was used to calculate the specific surface areas (*S*), and specific pore volumes (*V*_p_) and the average pore diameter (*d*_p_) were measured according to the BJH theory. Scanning electron microscopy (SEM) images were obtained on a scanning electron microscope (JEOL JSM-6490LV, Tokyo, Japan). The conditions were as follows: acceleration voltage, 0.5–30 kV; magnification, 10–10^6^. The samples were coated with thin layers of gold to make the surface conductive. Transmission electron microscopy images of mesoporous material films were observed by a transmission electron microscope (JEM 2100, Tokyo, Japan) at an operating voltage of 200 kV.

### 2.4. Adsorption Experiments

An amount of 100 mL crystal violet solution was added to each conical flask, together with 0.03 g of mesoporous material. After oscillating at 120 r/min for a certain time, filtration was carried out with 0.45 μm microporous membrane. During the adsorption process, the concentration of the dye solution was obtained by measuring the absorbance of the solution using the control variable method by UV/vis spectrophotometer (Lambda25, Perkin Elmer, Waltham, MA, USA), and the adsorption rate and adsorption quantity were calculated [18]. Thus, the adsorption equilibrium curve of each factor on the adsorption quantity of the molecular sieve could be obtained.

The adsorption rate of MCM-41 was calculated using Equation (1):

(1) $$r=\frac{C_{0}-C_{t}}{C_{0}}\times 100\%$$

where *C*_0_ is the initial dye concentration (mg/L) and *C_t_* represents the dye concentration of the adsorbate after time *t* (mg/L).

The adsorbed amount of dye was calculated from Equation (2):

(2) $$q_{t}=\frac{\left[\left(C_{0}-C_{t}\right)\times \frac{V}{1000}\right]}{m}$$

where *q_t_* represents the amount adsorbed after time *t* (mg/g), *m* is the weight of the MCM-41 used (g) and *V* denotes the volume of the solution (mL).

## 3. Results and Discussion

### 3.1. The ^1^H-NMR and ^13^C-NMR Analysis of [N_1116_C_3_IMC_16_][Br]_2_

The ^1^H NMR spectrum of [N_1116_C_3_C_16_IM][Br]_2_: 0.84~0.87 (td, 6H, 1, 40-CH_3_), 1.24~1.28 (m, 52H, 2~14, 27~39-CH_2_), 1.60~1.66 (m, 2H, 26-CH_2_), 1.78~1.83 (m, 2H, 15-CH_2_), 2.28~2.33 (m, 2H, 19-21CH_2_), 3.05 (s, 6H, 23, 24-CH_3_), 3.27~3.35 (m, 4H, 22, 25-CH_2_), 4.18~4.20 (t, 2H, 16-CH_2_), 4.26~4.29 (t, 2H, 20-CH_2_), 7.87 (t, 1H, 18-CH), 7.90~7.91 (t, 1H, 19-CH), 9.37 (s, 1H, 17-CH). 

^13^C NMR spectrum of [N_1116_C_3_C_16_IM][Br]_2_: 14.42 (1, 40-CH_3_), 22.19 (2, 39-CH_2_), 22.58 (3, 38-CH_2_), 23.28 (4, 37-CH_2_), 26.05 (5, 3236-CH_2_), 26.28 (6, 35-CH_2_), 28.95~29.82 (7~15, 26~34-CH_2_), 31.78 (21-CH_2_), 46.47 (16-CH_2_), 49.42 (23, 24-CH_3_), 50.66 (20-CH_2_), 59.99 (22-CH_2_), 63.68 (25-CH_2_), 122.92 (18-CH), 123.02 (19-CH), 136.78 (17-CH).

^1^H NMR and ^13^C NMR spectroscopy confirmed the chemical structure of the synthesized [N_1116_C_3_C_16_IM][Br]_2_.

### 3.2. Structural Characterization of Mesoporous Molecular Sieves

#### 3.2.1. FTIR Spectroscopy Analysis

**Figure 4:**
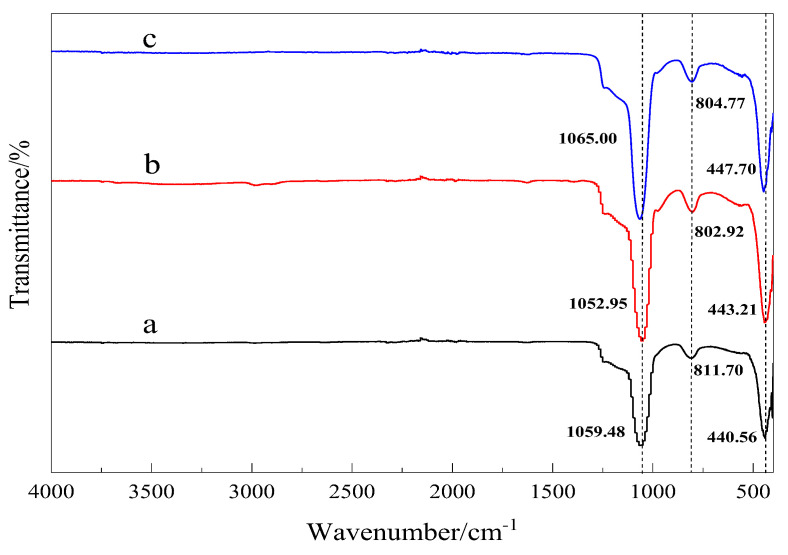
FTIR spectra for the samples. **a**—Mesoporous material synthesized with Gemini ionic liquid surfactant [N_1116_C_3_IMC_16_][Br]_2_ as template. **b**—Mesoporous material synthesized with Gemini ionic liquid surfactant [N_1116_C_3_N_1116_][Br]_2_ as template. **c**—Mesoporous material synthesized with Gemini ionic liquid surfactant [C_16_IMC_3_C_16_IM][Br]_2_ as template.

Figure 4 shows the FTIR spectra of MCM-41 prepared from [N_1116_C_3_IMC_16_][Br]_2_, [N_1116_C_3_N_1116_][Br]_2_ and [C_16_IMC_3_C_16_IM][Br]_2_. For sample a, the signals at 1051–1060 cm^−1^ are attributed to asymmetric vibrations of Si-O-Si. The signals at 802–811 cm^−1^ are attributed to symmetric stretching vibrations of Si-O-Si and the signals at 439–443 cm^−1^ are attributed to the bending vibration of Si-O-Si, which is consistent with the results reported in the literature [19,20]. The FTIR spectra of three samples were all determined, which indicated that the synthesized samples were all silica.

#### 3.2.2. XPS Analysis

**Figure 5:**
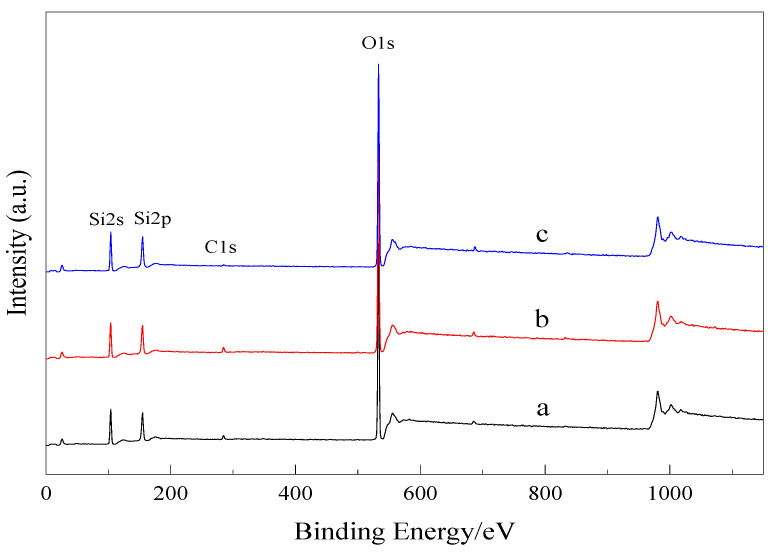
X-ray photoelectron spectroscopy for the samples. **a**—Mesoporous material synthesized with Gemini ionic liquid surfactant [N_1116_C_3_IMC_16_][Br]_2_ as template. **b**—Mesoporous material synthesized with Gemini ionic liquid surfactant [N_1116_C_3_N_1116_][Br]_2_ as template. **c**—Mesoporous material synthesized with Gemini ionic liquid surfactant [C_16_IMC_3_C_16_IM][Br]_2_ as template.

XPS analyses were performed to gain insight into the surface chemistry. The XPS spectra of the synthesized mesoporous molecular sieve samples are shown in Figure 5, and the analysis results are shown in Table 2. It can be seen that the sample exhibits major photoelectron peaks O1s (532.6 eV), Si2p (103.3 eV). In addition to Si2p (∼103 eV) and O1s (∼533 eV), it exhibits a very low intensity C1s peak due to adventitious hydrocarbon contamination. These results show that the synthesized material is silica. Similar characterization results were obtained for samples b and c. The results of XPS analysis show that element composition of the samples was identical and the synthesized material is silica, which is consistent with the FTIR analysis results.

#### 3.2.3. X-ray Diffraction

**Figure 6:**
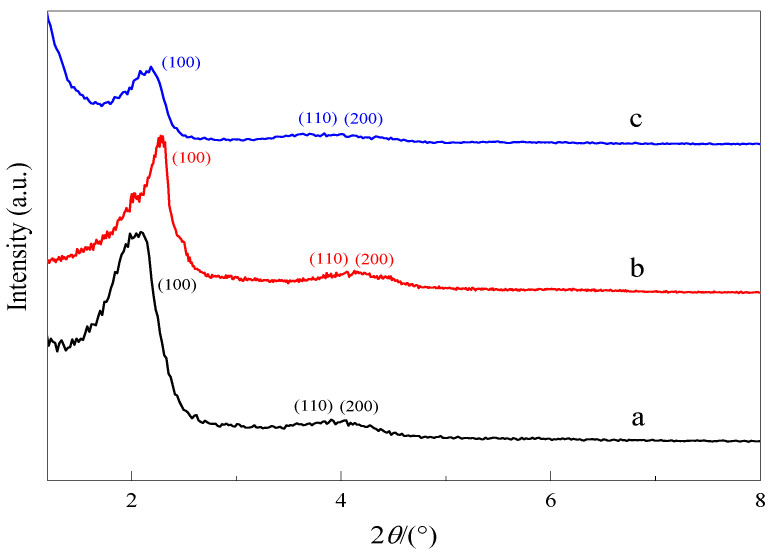
X-ray diffraction patterns of the samples. **a**—Mesoporous material synthesized with Gemini ionic liquid surfactant [N_1116_C_3_IMC_16_][Br]_2_ as template. **b**—Mesoporous material synthesized with Gemini ionic liquid surfactant [N_1116_C_3_N_1116_][Br]_2_ as template. **c**—Mesoporous material synthesized with Gemini ionic liquid surfactant [C_16_IMC_3_C_16_IM][Br]_2_ as template.

Small-angle X-ray diffraction spectra of the synthesized mesoporous molecular sieve samples are shown in Figure 6. The characteristic diffraction peaks were obtained for samples a, b and c. Three diffraction peaks can be seen at low Bragg angles: a very sharp maximum at 2°–2.5° and two additional reflections with lower intensities at 3.5°–4.5°. These peaks correspond, respectively, to the (100), (110) and (200) reflections. The analysis of small-angle X-ray diffraction outcomes can result in the following primary judgment: mesoporous silica MCM-41-type nanoparticles were synthesized using a wide variety of ionic liquids as the template. The intensities of the characteristic XRD peaks for MCM-41 decrease in intensity from sample a to sample c; this is because the crystalline phase content of mesoporous materials gradually decreases. The unit cell parameters of mesoporous materials were calculated from *a*_0_= 2*d_100_*/3^1/2^. According to the Bragg equation (2*d_100_*sin*θ* = *nλ*), where *d*, *θ*, *λ* and *n* are the interplanar spacing, diffraction angle, wavelength and indices of the crystal face, respectively, the results for specific surface area (*S*_BET_), pore volume (*V*_BJH_), unit cell size (*a*_0_), pore size (*D*_p_) and wall thickness (*Wt*) can be obtained, and are shown in Table 3.

As shown in Table 3, the specific surface area of mesoporous materials obtained by asymmetric Gemini ionic liquid surfactant [N_1116_C_3_IMC_16_][Br]_2_ as a template is larger than the specific surface area of mesoporous materials prepared by the other two kinds of surfactants as the template.

#### 3.2.4. Nitrogen Adsorption–Desorption Isotherms

**Figure 7:**
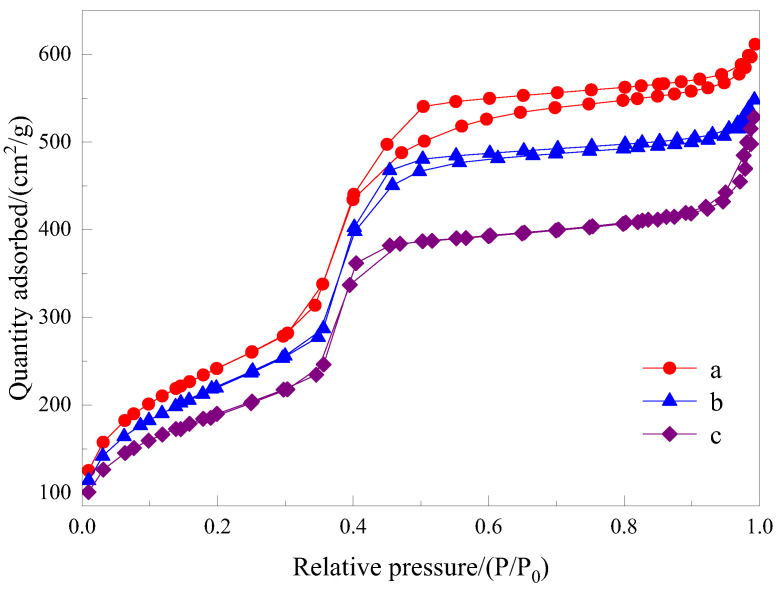
N_2_ adsorption–desorption isotherms of the samples. **a**—Mesoporous material synthesized with Gemini ionic liquid surfactant [N_1116_C_3_IMC_16_][Br]_2_ as template. **b**—Mesoporous material synthesized with Gemini ionic liquid surfactant [N_1116_C_3_N_1116_][Br]_2_ as template. **c**—Mesoporous material synthesized with Gemini ionic liquid surfactant [C_16_IMC_3_C_16_IM][Br]_2_ as template.

**Figure 8:**
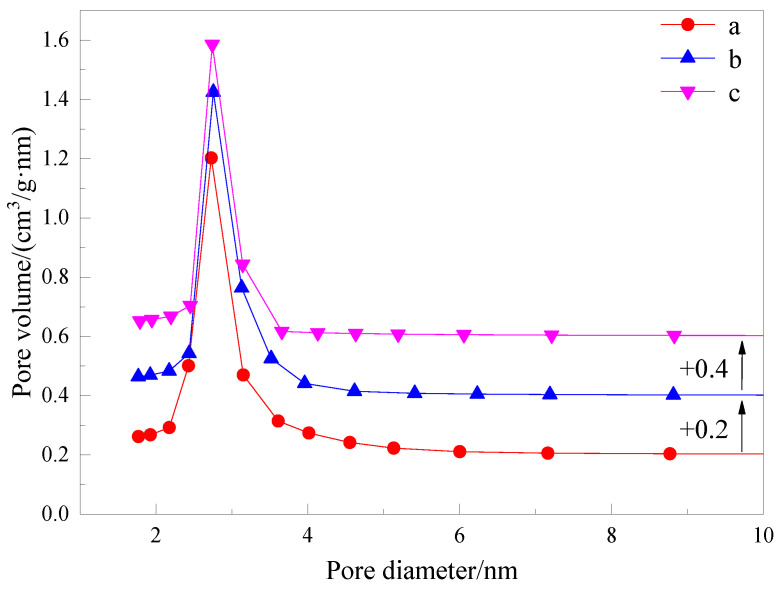
Pore size distribution of the samples. **a**—Mesoporous material synthesized with Gemini ionic liquid surfactant [N_1116_C_3_IMC_16_][Br]_2_ as template. **b**—Mesoporous material synthesized with Gemini ionic liquid surfactant [N_1116_C_3_N_1116_][Br]_2_ as template. **c**—Mesoporous material synthesized with Gemini ionic liquid surfactant [C_16_IMC_3_C_16_IM][Br]_2_ as template.

Nitrogen sorption isotherms of the samples are presented in Figure 7, and Figure 8 shows the pore size distribution curve of samples.

The N_2_ adsorption–desorption isotherm of samples demonstrates a typical IV-type isotherm containing H1-type hysteresis, suggesting a mesoporous structure. The adsorption and desorption curves overlap each other, which indicates that the obtained materials have an orderly structure, uniform pore size and good pore connectivity. The adsorption isotherm shows an obvious upward trend within the range of the relative pressure (0.3~0.4). The same typical type-IV adsorption–desorption isotherms were obtained for samples b and c, indicating that they also had the same structural characteristics. The pore structure parameters of samples are given in Table 3. According to the comparison of pore structure parameters of samples, mesoporous materials prepared by asymmetric Gemini ionic liquid surfactant [N_1116_C_3_IMC_16_][Br]_2_ have the largest specific surface area, while mesoporous materials prepared by symmetric imidazole ionic liquid have the largest wall thickness. The higher wall thickness of mesoporous materials can make the mesoporous structure more stable, while the larger specific surface area can provide greater dye adsorption efficiency. However, the material prepared by using asymmetric Gemini ionic liquid surfactant [N_1116_C_3_IMC_16_][Br]_2_ as a template has little difference in wall thickness compared with that prepared by using symmetric ionic liquid as a template, but the specific surface area is much larger than that of other materials, which gives the material synthesized by asymmetric ionic liquid more suitable structural characteristics for dye adsorption than the other two.

#### 3.2.5. Scanning and Transmission Electron Microscopy

**Figure 9:**
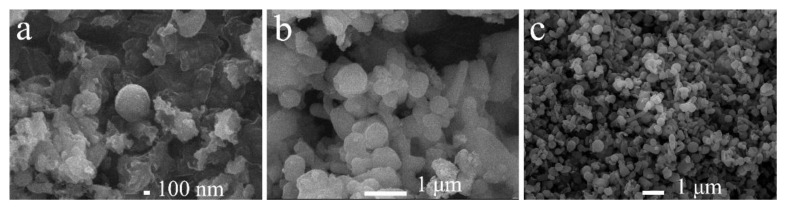
SEM images of the samples. (**a**) Mesoporous material synthesized with Gemini ionic liquid surfactant [N_1116_C_3_IMC_16_][Br]_2_ as template; (**b**) Mesoporous material synthesized with Gemini ionic liquid surfactant [N_1116_C_3_N_1116_][Br]_2_ as template; (**c**) Mesoporous material synthesized with Gemini ionic liquid surfactant [C_16_IMC_3_C_16_IM][Br]_2_ as template.

The morphology and shape of the mesoporous materials were characterized by scanning electron microscopy (Figure 9). The synthesized MCM-41 mesoporous silica particles have a well-defined spherical morphology; the particle size distribution is 1–2 μm.

**Figure 10:**
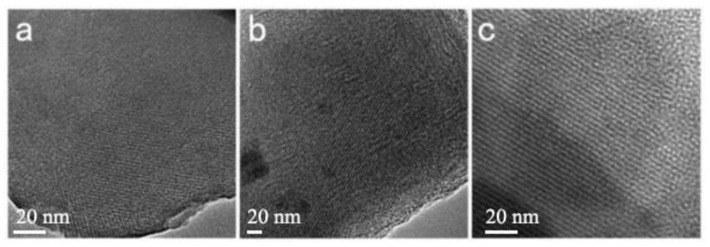
TEM images of the samples. (**a**) Mesoporous material synthesized with Gemini ionic liquid surfactant [N_1116_C_3_IMC_16_][Br]_2_ as template; (**b**) Mesoporous material synthesized with Gemini ionic liquid surfactant [N_1116_C_3_N_1116_][Br]_2_ as template; (**c**) Mesoporous material synthesized with Gemini ionic liquid surfactant [C_16_IMC_3_C_16_IM][Br]_2_ as template.

Transmission electron microscopy (TEM) images of the three samples are shown in Figure 10. Figure 10 shows that the three samples have clear (100) and (110) crystal planes of hexagonal phase, which proves that the samples have good two-dimensional hexagonal ordered pores. The sample pore size is about 3~4 nm. This result is consistent with the BJH test result. At the same time, the formation of mesoporous molecular sieves with the above structure shows that ionic liquids as templates form hexagonal micelles in solution.

### 3.3. Formation Mechanism of Mesoporous Molecular Sieves

**Figure 11:**
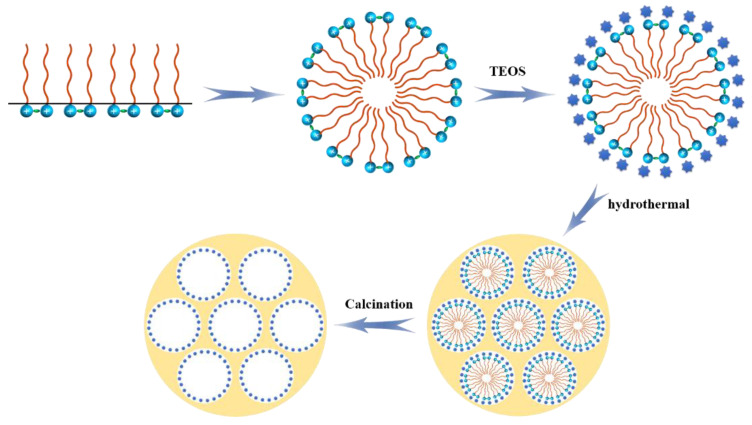
Schematic diagram of preparation of mesoporous molecular sieve with Gemini ionic liquid surfactant as templating agent.

Surfactant molecules are generally composed of nonpolar lipophilic groups (hydrophobic groups) and polar hydrophilic groups (oleophobic groups). Their structure determines that they have both hydrophilic and lipophilic properties. Gemini ionic liquid surfactant is a new type of surfactant. Its structure comprises two hydrophilic groups that are connected by connecting groups. Compared with traditional surfactants, Gemini ionic liquid surfactants have a lower critical micelle concentration, and as a template for mesoporous molecular sieve synthesis, it is easier to act as a template at a lower concentration. The mechanism of preparing a mesoporous molecular sieve with Gemini ionic liquid surfactant [21] is shown in Figure 11.

As shown in Figure 11, Gemini ionic liquid surfactant is amphiphilic, and is enriched on the solution surface when dissolved in water. At the same time, due to its unique micelle self-assembly behavior, it can form micelles in the solution as shown in the figure, which is the soft template for the formation of the mesoporous molecular sieve. Under alkaline conditions, tetraethyl orthosilicate is hydrolyzed in solution to form silica precursor silicate ions. Silicate ions are adsorbed to imidazolium cations through charge matching to form composites made of silica template aggregates. The mesoporous molecular sieve is formed by precipitation and aging at high temperature and high pressure, and the template is removed by calcination without damaging the formed mesoporous molecular sieve structure. Finally, the mesoporous molecular sieve with the expected structure is obtained.

### 3.4. Adsorption Properties of Mesoporous Material

Mesoporous molecular sieve sample a, prepared from asymmetric Gemini ionic liquid surfactant ([N_1116_C_3_IMC_16_][Br]_2_), was used as the adsorbent to explore the optimal adsorption conditions with crystal violet as the adsorbent, and its adsorption thermodynamics and kinetics were analyzed. 

#### 3.4.1. The Effect of Adsorption Time on the Adsorption Performance of Mesoporous Material

**Figure 12:**
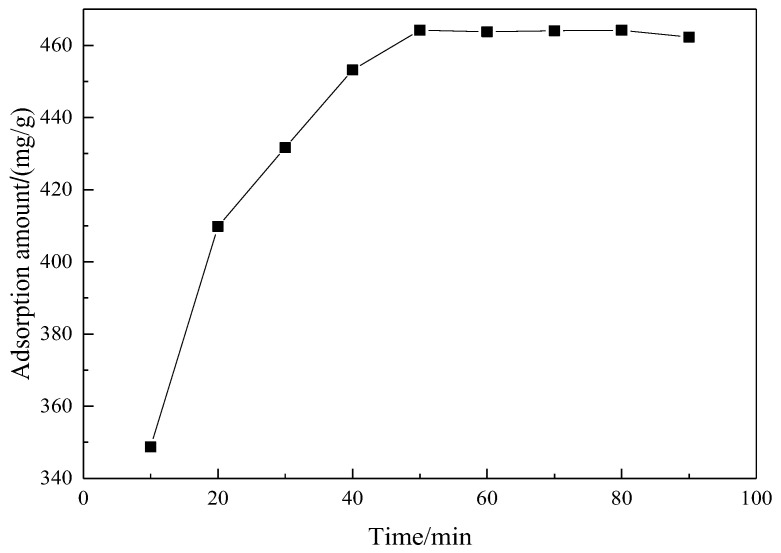
The influence of adsorption time on adsorption effect.

The amount of MCM-41 was 0.03 g and the adsorption temperature was kept constant at 35 °C. The curve of adsorption time and adsorption quantity is shown in Figure 12.

As shown in Figure 12, adsorption quantity increases with adsorption time, conforming to adsorption kinetics general laws. After 50 min, the adsorption process reached adsorption equilibrium and the adsorption quantity was 464.21 mg/g, higher than the data reported by Lee [10] and Li [15].

#### 3.4.2. The Effect of pH of Solution on the Adsorption Performance of Mesoporous Material

**Figure 13:**
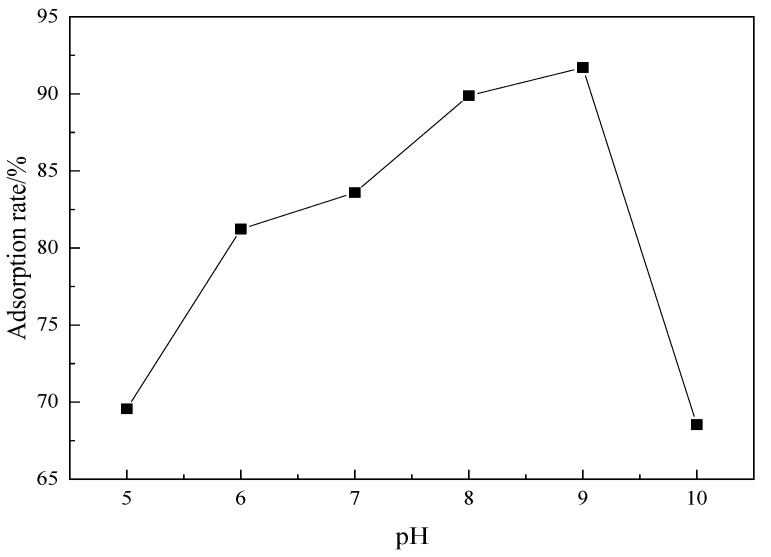
The influence of pH value on the adsorption rate.

The amount of MCM^-^41 was 0.03 g and the adsorption temperature was kept constant at 35 °C. The pH value of the adsorption solution was adjusted from 5 to 9 using HCl and NaOH. The curve of pH of solution and adsorption quantity is shown in Figure 13.

Figure 13 shows that the adsorption rate increases with the increase in pH value. Crystal violet is a cationic dye and H^+^ will compete with crystal violet adsorption sites in an acidic solution, affecting the adsorption of dye on the molecular sieve. With the increase in pH value, the concentration of H^+^ in solution decreases, and the pore and surface negative groups of the molecular sieve increase, increasing the adsorption rate. As shown in the Figure 13, when the pH value reaches 9, the adsorption effect of the molecular sieve reaches the maximum value. However, with the increase in pH value, the adsorption rate decreases sharply for the reason that the mesoporous structure is destroyed by the reaction between the molecular sieve and the alkali. Therefore, the optimal pH value of the solution should be 9.

#### 3.4.3. The Effect of Adsorption Temperature on the Adsorption Performance of Mesoporous Material

**Figure 14:**
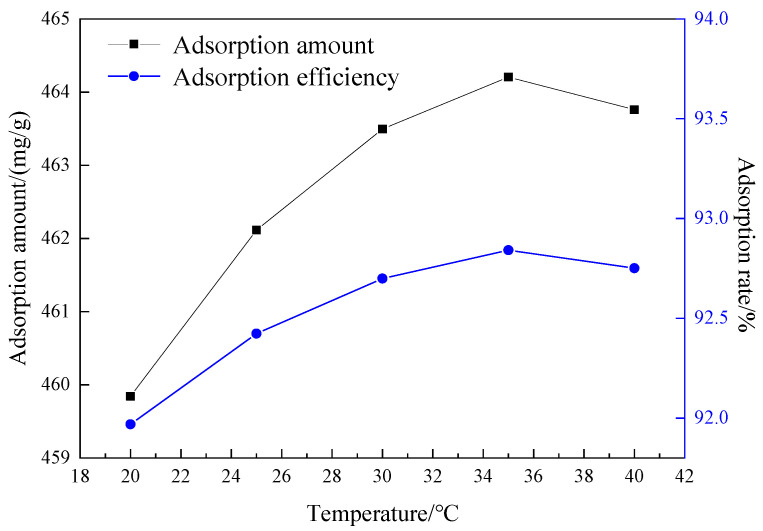
The influence of the temperature on the adsorption effect.

The effect of adsorption temperature on the adsorption performance of mesoporous material is shown in Figure 14 with the amount of MCM-41 0.03 g, the adsorption time 50 min and the pH value of the solution 9.

It can be seen from Figure 14 that the adsorption capacity and adsorption rate increase with the increase in temperature in the range 20~35 °C, which indicates that crystal violet adsorption by MCM-41 is an endothermic process within this temperature range, and the increase in temperature is conducive to the adsorption [22]. When the temperature is higher than 35 °C, the adsorption rate and adsorption capacity decrease as the temperature increases, indicating that the adsorption process is exothermic. The adsorption rate and adsorption capacity reach the highest levels (adsorption capacity 464.21 mg/g, adsorption rate 92.84%) at 35 °C. Therefore, the best adsorption temperature in the experiment was 35 °C.

#### 3.4.4. The Effect of Initial Concentration of Adsorbate on the Adsorption Performance of Mesoporous Material

**Figure 15:**
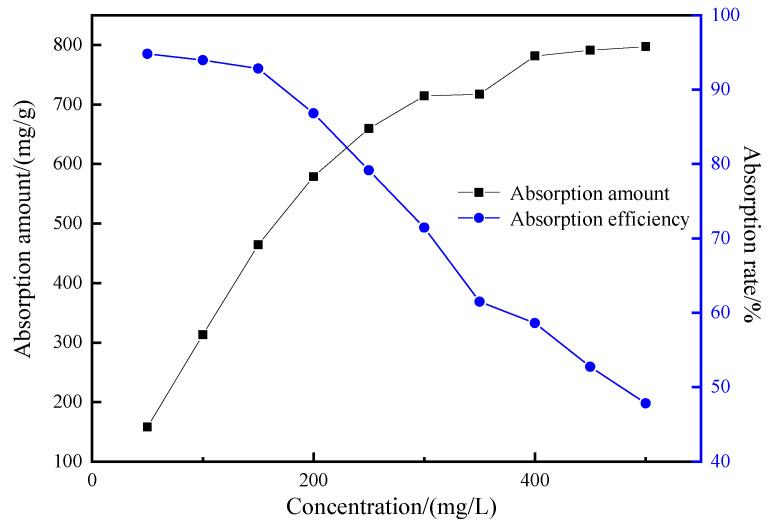
The influence of the initial mass concentration of adsorbate on the adsorption effect.

The effect of initial concentration of the adsorbate on the adsorption performance of the mesoporous material is shown in Figure 15 with the amount of MCM-41 0.03 g, the pH value of the solution 9, the adsorption temperature 35 °C and the adsorption time 50 min. 

As shown in Figure 15, with the increase in the initial concentration of adsorbent, the adsorption capacity increases. When the crystal violet concentration is 500 mg/g, the adsorption capacity can reach 797.24 mg/g, while the adsorption rate decreases. With the increase in dye concentration, the adsorption site is saturated and the adsorption is close to equilibrium. With the increase in concentration of dye, the adsorption site is gradually excessive, so the adsorption rate gradually decreases.

### 3.5. Analysis of Adsorption Kinetics and Adsorption Isotherm Regression Analysis of Mesoporous Materials

#### 3.5.1. Analysis of Adsorption Kinetics of Mesoporous Materials

According to the linear equation of the quasi-second-order adsorption kinetic model [16,23,24], we have the following:

(3) $$\frac{t}{q_{t}}=\frac{1}{k_{2}q_{e}^{2}}+\frac{1}{q_{e}}t$$

where *q_e_* represents the equilibrium adsorption capacity of adsorbent (mg/g) and *k*_2_ is the rate constant of the second-order adsorption (g/(mg·min)).

**Figure 16:**
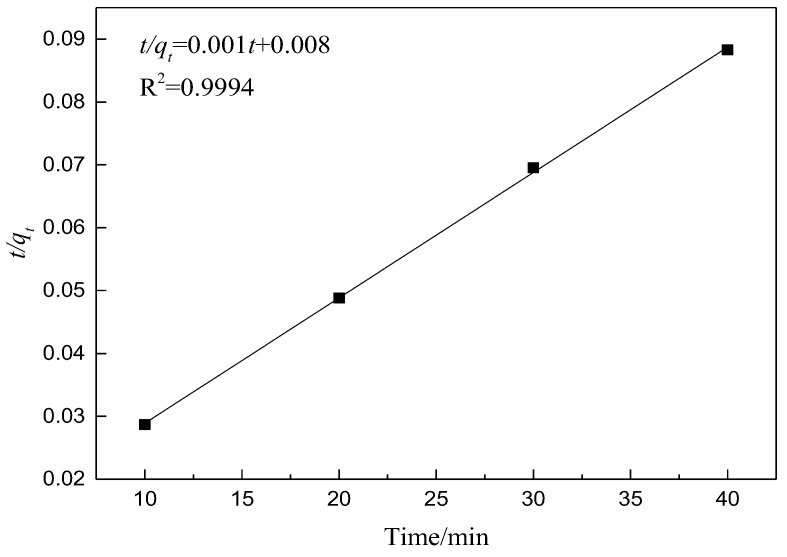
Regression curve of second-order rate equation.

*t*/*q_t_* is regarded as the ordinate and *t* as the abscissa. The relevant data of adsorption kinetics are shown in Table 4, from which it can be determined whether the adsorption conforms to the second-order adsorption kinetics according to the figure and relevant data. The second-order rate equation regression curve of the mesoporous molecular sieve adsorbing crystal violet is presented in Figure 16.

As seen in Figure 16, the correlation coefficient of the regression equation of the second-order adsorption rate is 0.9994, indicating that this process conforms to the second-order adsorption kinetics [12,25].

#### 3.5.2. Analysis of the Adsorption Isotherm Regression

The Langmuir model [12,25] was employed to analyze the isotherm regression of the adsorption of crystal violet on the mesoporous molecular sieve.

(4) $$\frac{1}{q_{e}}=\frac{1}{q_{m}}+\frac{1}{q_{m}K_{L}C_{e}}$$

where *q_m_* denotes the maximum adsorption capacity (mg/g) and *K_L_* is the equilibrium constant.

**Figure 17:**
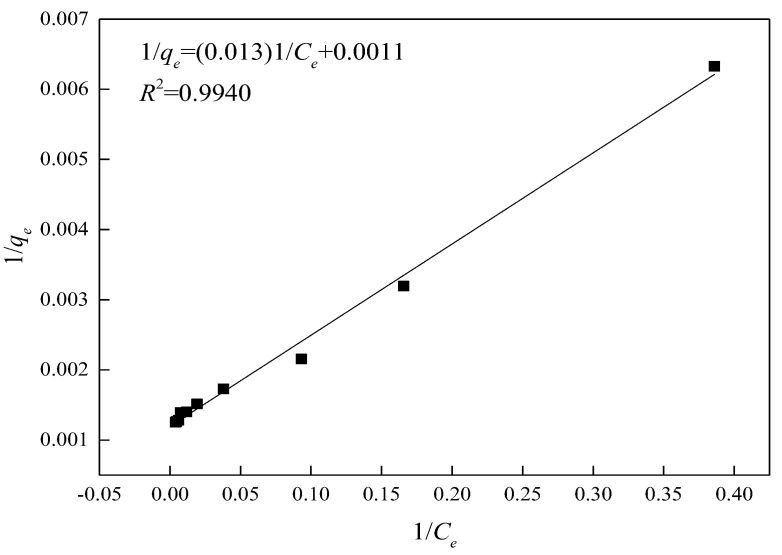
The regression curve of the Langmuir isotherm adsorption equation.

The relevant data are shown in Table 5 and the Langmuir isotherm is shown in Figure 17. The correlation coefficient R^2^ of the isothermal regression curve is 0.9940, indicating that the adsorption behavior of crystal violet on the mesoporous molecular sieve belongs to Langmuir adsorption, and the theoretical maximum adsorption capacity *q_m_* is up to 869.69 mg/g.

#### 3.5.3. Adsorption Thermodynamic Analysis

Thermodynamic equations:

(5) $$K_{d}=\frac{q_{e}}{C_{e}}$$

(6) $$\Delta G=-RT\ln K_{d}$$

(7) $$\ln K_{d}=-\frac{\Delta H}{RT}+\frac{\Delta S}{R}$$

(8) ΔG=ΔH−TΔS

where *R* is the ideal gas constant, 8.314 J/(mol·K), *T* represents the absolute temperature (K), Δ*G* is the adsorption free energy (kJ/mol), Δ*H* is the adsorption enthalpy (kJ/mol) and Δ*S* is the adsorption entropy (J/mol).

**Figure 18:**
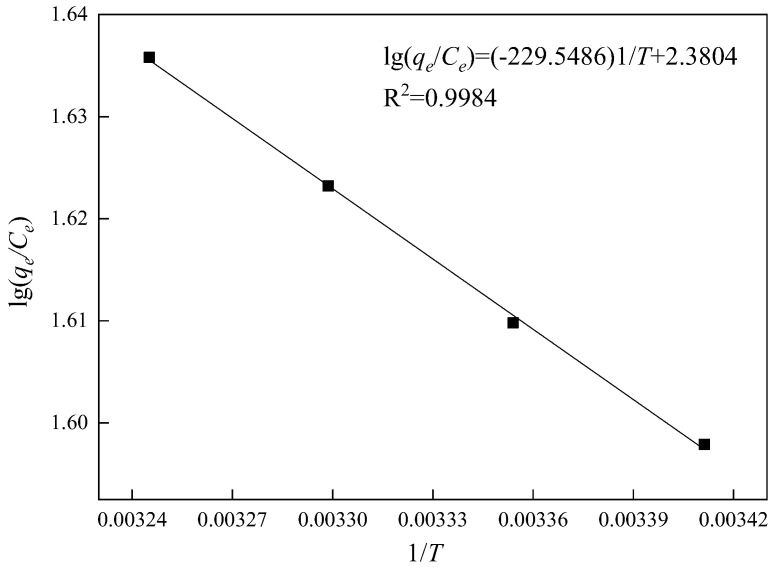
Relationship of lg(*q*_e_/*C*_e_) and 1/*T*.

The relationship between lg(*q_e_*/*C_e_*) and 1/*T* is given in Figure 18. The calculated thermodynamic data are shown in Table 6. According to Figure 18, the thermodynamic parameters were obtained, namely Δ*H* = −1.91 kJ/mol, Δ*S* = 19.79 J/mol and Δ*G* = −7.71 kJ/mol. Since both chemical adsorption and physical adsorption are spontaneous processes, the Gibbs free energy decreases during the adsorption process (Δ*G* < 0). When the adsorbate molecules are adsorbed on the solid surface, the adsorbate molecules move freely in space, so the degree of freedom of motion increases, and the entropy also increases (Δ*S* > 0), indicating that the adsorption process is a spontaneous process.

## 4. Conclusions

MCM-41 mesoporous molecular sieves with an ordered structure were successfully prepared by hydrothermal synthesis with various Gemini surfactants ([N_1116_C_3_IMC_16_][Br]_2_, [N_1116_C_3_N_1116_][Br]_2_, [C_16_IMC_3_C_16_IM][Br]_2_) as templates and were characterized by FTIR, XPS, SEM, TEM, XRD, etc. The results show that the mesoporous molecular sieves have a regular and orderly pore structure. The mesoporous material prepared by asymmetric Gemini ionic liquid surfactant ([N_1116_C_3_IMC_16_][Br]_2_) possesses larger specific surface area than the other surfactants, which gives the material synthesized by [N_1116_C_3_IMC_16_][Br]_2_ more suitable structural characteristics for dye adsorption than the other two. The results of adsorption experiments on mesoporous materials show that the mesoporous molecular sieve MCM-41 prepared by [N_1116_C_3_IMC_16_][Br]_2_ has a good adsorption effect on crystal violet, and the adsorption capacity can reach 464.21 mg/g, indicating that the synthesized mesoporous molecular sieve has excellent adsorption performance for dyes. The optimum conditions (pH = 9, adsorption temperature 35 °C, adsorption time 50 min) for mesoporous molecular sieve MCM-41 adsorbing crystal violet were obtained by the static adsorption method. The analysis of adsorption kinetics of mesoporous materials shows that the correlation coefficient of the regression equation of the second-order adsorption rate is 0.9994, indicating that this process conforms to second-order adsorption kinetics. The analysis of the adsorption isotherm regression indicates that the adsorption behavior of crystal violet on the mesoporous molecular sieve belongs to Langmuir adsorption. The analysis of adsorption thermodynamics indicates that the adsorption process is a spontaneous process.

## Figures and Tables

**Table 1:** Conditions used in the preparation of MCM-41.

Sample	Reactants	Molar Ratio	Mass Ratio
a	TEOS/[N_1116_C_3_IMC_16_][Br]_2_/H_2_O	1.0:0.1:100.0	2.08/0.79/17.97
b	TEOS/[N_1116_C_3_N_1116_][Br]_2_/H_2_O	1.0:0.1:100.0	2.08/0.74/17.97
c	TEOS/[C_16_IMC_3_C_16_IM][Br]_2_/H_2_O	1.0:0.1:100.0	2.08/0.79/17.97

**Table 2:** XPS element analysis results of samples.

Sample	Name	Peak Position/eV	Full Width at Half Maximum /(FWHM)	Atomic Concentration/%
a	Si2p	103.96	1.54	12.79
	O1s	533.31	1.49	84.97
	C1s	284.88	1.53	2.24
b	Si2p	103.84	1.52	12.60
	O1s	533.22	1.44	84.92
	C1s	284.82	1.42	2.48
c	Si2p	103.93	2.75	31.42
	O1s	533.14	2.73	64.52
	C1s	285.32	4.19	2.34

**Table 3:** Pore structure parameters of samples.

Sample	*S*_BET_/(m^2^/g)	*V*_BJH_/(cm^3^/g)	*a*_0_/nm	*D*_p_/nm	*Wt*/nm
a	879.37	0.85	3.88	3.28	0.90
b	803.01	0.95	4.23	3.32	0.59
c	684.08	0.82	4.78	3.86	0.92

**Table 4:** Adsorption kinetic data of crystal violet on MCM-41.

*t*/min	*C_e_*/(mg/L)	*q_t_*/(mg/g)	*t/q_t_*
10	45.38	348.73	0.0287
20	27.05	409.83	0.0488
30	20.51	431.63	0.0695
40	14.05	453.16	0.0883
50	10.74	464.21	
60	10.87	463.76	
70	10.79	464.02	
80	10.74	464.21	
90	11.32	462.26	

**Table 5:** Adsorption isotherm data of crystal violet on MCM-41.

*C*_0_/(mg/L)	*C_e_*/(mg/L)	*q_e_*/(mg/g)	1/*C_e_*	1/*q_e_*
50	2.59	158.03	0.3861	0.0063
100	6.04	313.21	0.1657	0.0032
150	10.74	464.21	0.0931	0.0022
200	26.34	578.88	0.0380	0.0017
250	52.12	659.61	0.0192	0.0015
300	85.63	714.57	0.0117	0.0014
350	134.77	717.43	0.0074	0.0014
400	165.51	781.62	0.0060	0.0013
450	212.64	791.18	0.0047	0.0013
500	260.83	797.24	0.0038	0.0013

**Table 6:** Adsorption thermodynamic data of MCM-41.

*T*/K	*C_e_* (mg/L)	*q_e_* (mg/g)	*q_e_/C_e_*	lg(*q_e_/C_e_*)
293.15	11.65	461.17	39.59	1.60
298.15	11.35	462.17	40.72	1.61
303.15	11.03	463.23	41.99	1.62
308.15	10.74	464.21	43.23	1.64

## Data Availability

Data sharing is not applicable.
